# Development of PET and SPECT Probes for Glutamate Receptors

**DOI:** 10.1155/2015/716514

**Published:** 2015-03-22

**Authors:** Takeshi Fuchigami, Morio Nakayama, Sakura Yoshida

**Affiliations:** Department of Hygienic Chemistry, Graduate School of Biomedical Sciences, Nagasaki University, 1-14 Bunkyo-machi, Nagasaki 852-8521, Japan

## Abstract

l-Glutamate and its receptors (GluRs) play a key role in excitatory neurotransmission within the mammalian central nervous system (CNS). Impaired regulation of GluRs has also been implicated in various neurological disorders. GluRs are classified into two major groups: ionotropic GluRs (iGluRs), which are ligand-gated ion channels, and metabotropic GluRs (mGluRs), which are coupled to heterotrimeric guanosine nucleotide binding proteins (G-proteins). Positron emission tomography (PET) and single photon emission computed tomography (SPECT) imaging of GluRs could provide a novel view of CNS function and of a range of brain disorders, potentially leading to the development of new drug therapies. Although no satisfactory imaging agents have yet been developed for iGluRs, several PET ligands for mGluRs have been successfully employed in clinical studies. This paper reviews current progress towards the development of PET and SPECT probes for GluRs.

## 1. Introduction

l-Glutamate is the primary endogenous excitatory neurotransmitter and glutamate receptors (GluRs) are implicated in a range of neurological functions within the mammalian central nervous system (CNS). Two distinct groups of GluRs have been identified: ionotropic receptors (iGluRs) and metabotropic receptors (mGluRs). iGluRs form ligand-gated ion channels and are classified into three subtypes based on their pharmacological properties: NMDA (*N*-methyl-d-aspartate receptors, NMDARs), AMPA (*α*-amino-3-hydroxy-5-methylisoxazole-4-proprionic acid) receptors, and kainate receptors [1]. mGluRs are coupled to heterotrimeric guanosine nucleotide binding proteins (G-proteins) and include eight receptor subtypes, classified into three groups according to their sequence homology, signal transduction, and pharmacological profiles; group I includes mGluR1 and mGluR5, group II includes mGluR2 and mGluR3, and group III includes mGluR4, mGluR6, mGluR7, and mGluR8 [2]. Impaired regulation of GluRs may be involved in the pathophysiology of various brain disorders [3, 4]. Positron emission tomography (PET) and single photon emission computed tomography (SPECT) imaging for GluRs are considered to be powerful tools for the evaluation of excitatory neurotransmission in the living brain, the study of the pathophysiology of related neurological disorders, and the quantification of GluR drug occupancy* in vivo*. To date, no specific radioligands for* in vivo* visualization of iGluRs have been identified. In contrast, there are several promising clinically useful PET ligands for mGluRs. This review summarizes current progress towards the development of PET and SPECT probes for GluRs, with a particular focus on NMDARs and mGluRs.

## 2. NMDARs

The NMDARs are iGluRs that play key roles in processes involving excitatory neurotransmission, including learning, memory, and synaptic plasticity [5, 6]. Dysregulation of NMDARs has been identified in various neurological diseases, including epilepsy, ischemia, stroke, Parkinson's disease, Alzheimer's disease, Huntington's disease, and schizophrenia [4, 7, 8]. Activation of these receptors requires binding of glutamate and glycine and removal of Mg^2+^ blockade by membrane depolarization. NMDAR channel opening results in calcium influx into cells, followed by Ca^2+^-dependent signal transduction cascades that modulate many aspects of neuronal function [1, 9]. Functional NMDARs are composed of two NR1 subunits, together with either two NR2 subunits or a combination of NR2 and NR3 subunits [10–12]. NR1 subunits are ubiquitously distributed throughout the brain. There are four types of the NR2 subunit (NR2A, NR2B, NR2C, and NR2D), with distinct distributions in the brain [13]. NR3 subunits can be activated by glycine alone. NR3A is expressed in the cortex and brainstem, while NR3B is distributed in the forebrain and cerebellum [14]. The glutamate binding site is present on the NR2 subunit, whereas the glycine binding site is located on NR1 or NR3 [11, 15, 16]. Polyamines are allosteric potentiators of NMDARs containing NR2B subunits, presumably through recognition of the NR1 and NR2B dimer interface [17]. It is known that aliphatic cyclic amine groups strongly inhibit NMDAR activity in a noncompetitive and voltage-dependent manner [18, 19]. Negative modulators of NR2B, including ifenprodil and its derivatives, have been found to bind at the interface between the NR1 and NR2B subunits [20]. The main strategy for development of PET and SPECT ligands is the structural modification of NMDA receptor antagonists, including channel blockers, glycine site antagonists, and NR2B negative modulators. Because most competitive antagonists of the glutamate binding site have shown low selectivity and poor blood-brain barrier (BBB) permeability [1, 21], there are no reported radioligands interacting with this region of the NMDAR.

### 2.1. Imaging Probes for the NMDAR Channel Blocker Binding Site

**Figure 1:**
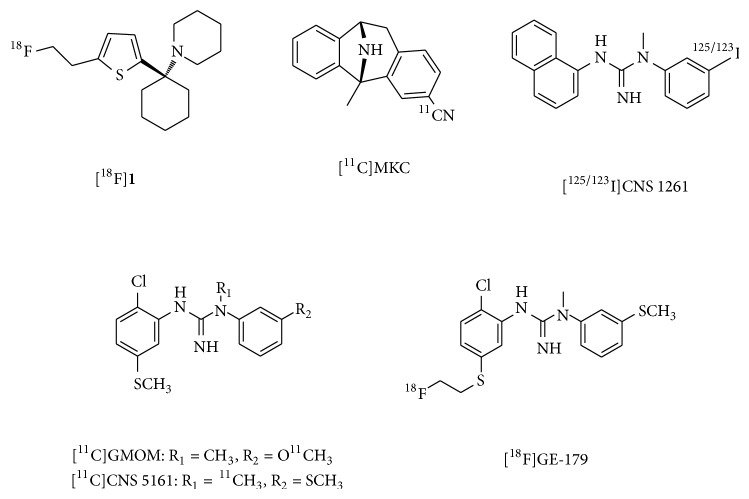
Chemical structure of imaging probes for channel blocker binding site of NMDARs.

**Figure 2:**
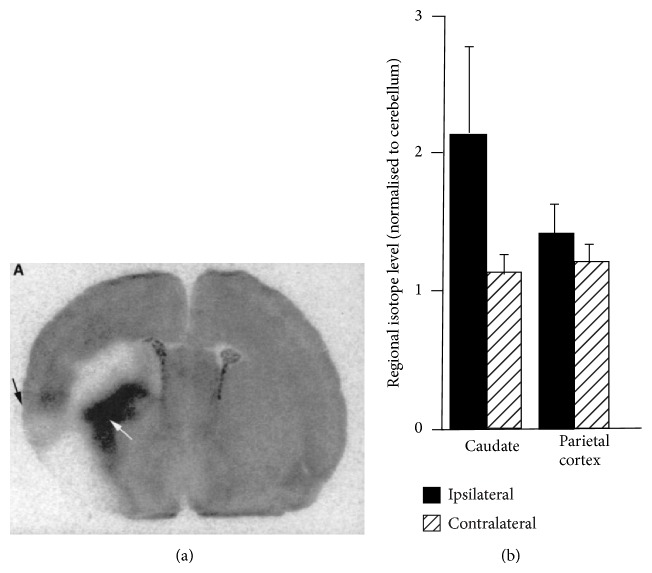
*Ex vivo *autoradiograms of [^125^I]CNS 1261 in the caudate nucleus (white arrow) and cerebral cortex (black arrow) of rat brain (a). Quantified regional isotope levels normalized to cerebellum (b). The animals were injected with [^125^I]CNS 1261 15 min after permanent occlusion of the middle cerebral artery (left hemisphere) and sacrificed 120 min later [26].

Open channel blockers of NMDARs, such as (+)-10,11-dihydro-5-methyl-5H-dibenzo[a,d] cyclohepten-5,10-diyldiammonium maleate (MK-801) and phencyclidine (PCP) derivatives, have been reported to bind to NMDARs in an activation-dependent manner [22, 23]. Thus, numerous* in vivo* imaging agents have been developed to interact with the PCP binding site, as this enables evaluation of the distribution of functional NMDARs in the brain under normal and pathological states. These agents include PCP, MK-801, ketamine, memantine, and diarylguanidine derivatives. [^18^F]**1** (Figure 1), a PCP derivative with an IC_50_ of 61 nM for the ion channel site, showed an* in vivo* distribution that was consistent with NMDAR expression. Furthermore, coinjection of 1.7 *μ*mol/kg of the high-affinity ion channel blocker,* cis*-2-hydroxymethyl-r-1-(*N*-piperidyl)-1-(2-thienyl)cyclohexane (*cis*-HPTC), resulted in a reduction in the regional cerebral distribution of [^18^F]**1**. However, this tracer was unsuitable for use as an NMDAR PET radioligand because of its high nonspecific binding in the brain [24]. A 3-[^11^C]cyano analog of MK-801 ([^11^C]MKC, Figure 1) has been reported as a PET ligand with excellent affinity for the channel blocker site (*K*_*d*_ = 8.2 nM). This tracer showed highly specific binding and heterogeneous* in vitro* distribution in rat brain slices that was similar to the expression of NMDARs. In PET studies, [^11^C]MKC showed a rapid and high uptake into the brains of rhesus monkeys, with higher accumulation in the frontal cortex than in the cerebellar cortex. However, these distribution patterns correlated closely with regional cerebral blood flow and blocking with NMDAR antagonists did not affect the regional brain distribution of this tracer [25]. PCP and MK-801 analogs showed high nonspecific* in vivo* binding, probably due to their high lipophilicity. Diarylguanidines have been identified as highly potent NMDAR channel blockers, with less hydrophobicity than PCP and MK-801. Therefore, several radiolabeled diarylguanidine analogs have been reported as PET or SPECT ligands.* N*-(1-naphthyl)-*N*′-(3-iodophenyl)-*N*′-methylguanidine (CNS 1261, Figure 1) has been developed as a high-affinity SPECT ligand for the ion channel site (*K*_*i*_ = 4.2 nM) with moderate lipophilicity (log*D* = 2.19). In* ex vivo* autoradiographic studies, [^125^I]CNS 1261 showed 2.4–2.9-fold higher uptake by the hippocampus than by the cerebellum in normal rat brains. This accumulation pattern was consistent with the pattern of NMDAR expression. In addition, investigation of [^125^I]CNS 1261 binding in a mouse model of cerebral ischemia revealed that [^125^I]CNS 1261 showed 2-fold higher uptake by the caudate nucleus in the ischemic hemisphere, as compared to the same region of the nonischemic hemisphere (Figure 2). This suggested that [^125^I]CNS 1261 bound selectively to activated NMDARs [26]. Based on this positive result, several clinical SPECT studies employing [^123^I]CNS 1261 have been performed. In healthy volunteers, no significant difference in the total distribution volume (*V*_*T*_) was observed between the NMDAR-rich regions (striatum, hippocampus, and frontal cortex) and the NMDAR-poor cerebellum [27, 28]. Numerous reports have suggested that hypofunction of NMDARs is associated with the pathophysiology of schizophrenia [29, 30]. It is reported that drug-free patients with schizophrenia showed reduced binding of [^123^I]CNS 1261 in the left hippocampus relative to the whole cortex, compared with healthy controls [31]. In contrast, a separate study demonstrated that *V*_*T*_ values of [^123^I]CNS 1261 in drug-free or typical antipsychotic-treated schizophrenia patients did not differ significantly from those observed in the control group [32]. Therefore, these reports did not provide evidence to support the proposal that NMDARs could be imaged by SPECT using [^123^I]CNS 1261.* N*-(2-chloro-5-thiomethylphenyl)-*N*′-(3-[^11^C]methoxy-phenyl)-*N*′-methylguanidine [^11^C]GMOM (Figure 1) is a ^11^C-labeled diarylguanidine derivative with a high affinity for the ion channel site (*K*_*i*_ = 5.2 nM). In PET studies conducted in baboons, [^11^C]GMOM showed BBB permeability. However, brain distribution of [^11^C]GMOM was almost homogeneous and preadministration of MK801 did not significantly change the regional *V*_*T*_ [33]. Another diarylguanidine derivative,* N*-(2-chloro-5-(methylmercapto)phenyl)-*N*′-[^11^C]methylguanidine monohydrochloride ([^11^C]CNS 5161, Figure 1), had excellent affinity for the ion channel site (*K*_*i*_ = 1.9 nM). [^3^H]CNS 5161 showed a heterogeneous* in vivo* distribution in rat brain and a cortex/cerebellum ratio of 1.4. Pretreatment with NMDA increased the hippocampus/cerebellum ratio to 1.6–1.9, while MK801 reduced the ratios to close to 1.0 [34]. Clinical PET studies using [^11^C]CNS 5161 indicated that the largest uptake occurred in the putamen and thalamus and the lowest uptake was observed in the cerebellum, but relatively low levels of radioactivity were detected in the NMDAR-rich hippocampus [35]. Further investigations are necessary in order to provide consistent evidence that these diarylguanidines can be used as PET or SPECT radioligands for the channel blocker binding site of the NMDAR. Recently, [^18^F]GE-179 (Figure 1), a high-affinity channel blocker (*K*_*i*_ = 2.4 nM) [36], was radiolabeled and used for PET imaging in healthy human subjects. Although this tracer showed high brain uptake, the *V*_*T*_ of each region was correlated to cerebral blood flow rather than the levels of NMDAR expression. Further characterization of [^18^F]GE-179 may be necessary with* in vivo* PET studies using NMDAR-activated models [37].

### 2.2. Imaging Probes for the NMDAR Glycine Binding Site

**Figure 3:**
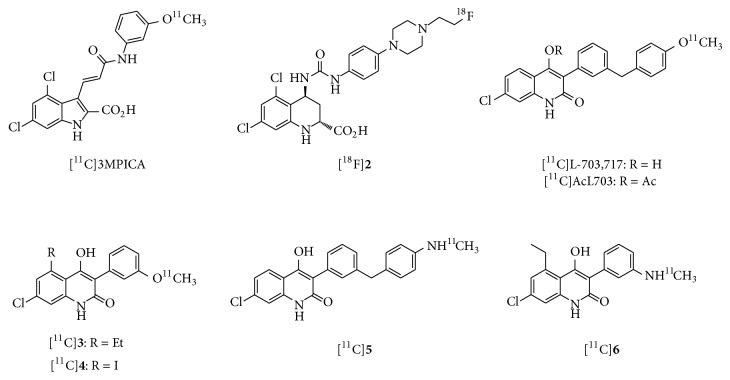
Chemical structure of imaging probes for glycine binding site of NMDARs.

**Figure 4:**
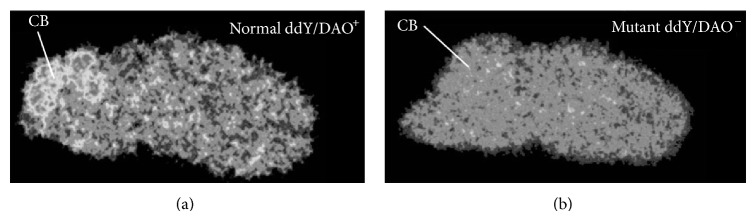
*Ex vivo *autoradiograms of [^11^C]L-703,717 in the brain of normal ddY/DAO+ (a) and mutant ddY/DAO-mice (b) 30 min after injection of [^11^C]L-703,717 and warfarin (60 mg/kg) [51].

A number of antagonists of the NMDAR glycine binding site have been developed as anticonvulsant and neuroprotective drugs [38]. Several radiolabeled cyclic amino acid derivatives, such as [^11^C]-3-[2-[(3-methoxyphenylamino) carbonyl]ethenyl]-4,6-dichloroindole-2-carboxylic acid ([^11^C]3MPICA, Figure 3) and [^18^F]**2**, have excellent binding affinities for the glycine binding site (*K*_*i*_ = 4.8 and 6.0 nM, resp.). However, they showed poor* in vivo* BBB permeability and had brain accumulation patterns that were inconsistent with those of the NMDAR [39, 40]. Since the low brain uptake of the cyclic amino acid derivatives was due to the highly polar charged carboxylate group, 4-hydroxyquinolones (4-HQs), which are carboxylic bioisosteres, have been investigated as high-affinity antagonists of the glycine binding site. 3-[3-(4-[^11^C]methoxybenzyl)phenyl]-4-hydroxy-7-chloroquinolin-2(1H)-one ([^11^C]L-703,717, Figure 3) has been developed as one of the most potent glycine site antagonists with a 4-HQ backbone (IC_50_ = 4.5 nM versus [^3^H]L-689,560) [41, 42].* In vivo* experiments in mice showed poor initial brain uptake of [^11^C]L-703,717 {0.32−0.36 percent injected dose per gram of tissue (% ID/g) at 1 min} and high levels of radioactivity in the blood. Since warfarin administration caused a dose-dependent enhancement of the initial brain uptake of [^11^C]L-703,717, this tracer may have a high affinity for plasma protein warfarin binding sites. The accumulation of [^11^C]L-703,717 in the cerebrum was lower than that observed in the cerebellum at 30 min (0.20% ID/g versus 0.65% ID/g). This distribution pattern was inconsistent with that of NMDAR expression. It should be noted that treatment with nonradioactive L-703,717 (2 mg/kg) only led to a significant reduction in the accumulation of [^11^C]L-703,717 in the cerebellum [43]. In order to improve the BBB permeability of [^11^C]L-703,717, an acetyl derivative of L-703,717 ([^11^C]AcL703, Figure 3) was developed as a prodrug radioligand. Initial brain uptake of [^11^C]AcL703 at 1 min was 2-fold higher than that of [^11^C]L-703,717. In rat brain tissues, approximately 80% of [^11^C]AcL703had been metabolized to [^11^C]L-703,717 by 20 min after injection. In* ex vivo* studies, [^11^C]AcL703 showed higher uptake in the cerebellum than in the cerebrum, consistent with the findings using [^11^C]L-703,717 [44]. Although a clinical PET study of [^11^C]AcL703 was performed in healthy volunteers, cerebellar NMDARs could not be visualized by PET due to poor BBB penetration [45]. Other radiolabeled 4-HQs (**3** and** 4**, Figure 3) with lower lipophilicity than [^11^C]L-703,717 have been developed as high-affinity radioligands for the glycine site (*K*_*i*_ = 7.2 and 10.3 nM). However, [^11^C]**3** and [^11^C]**4** did not exhibit a significant increase in brain uptake, as compared with [^11^C]L-703,717 [46]. The 4-HQs are acidic (p*K*_*a*_ ≦ 5) [42] and this may result in strong binding affinity for serum albumin and low BBB penetration. Thus, several amino 4-HQ derivatives with lower p*K*_*a*_ values were synthesized and evaluated as new PET radioligands for the glycine site. Methylamino derivatives of 4-HQs,** 5** and** 6** (Figure 3), showed high affinity for the glycine site (*K*_*i*_ = 11.7 nM and 11.8 nM, resp.). Although the amine derivatives showed a much lower plasma protein binding ratio than the methoxy analogs, [^11^C]**6** still displayed poor uptake into the brain [47]. Further structure-activity relationship studies are necessary to develop PET ligands for the glycine site with significantly improved BBB penetration. Furthermore, the brain distribution of imaging agents interacting with the glycine site can be greatly influenced by endogenous agonists. The NMDAR coagonists, glycine and d-serine, are present in the brain at micromolar levels. Glycine is ubiquitously distributed in the brain, while d-serine is predominantly found in the forebrain [48, 49]. Levels of d-serine are reportedly very low level in the cerebellum, because of the high expression level of an enzyme (d-amino acid oxidase, DAO) that can degrade d-serine [50]. Consistent with the above reports, the [^11^C]L-703,717 signal in the cerebellum was diminished in mutant ddY/DAO-mice, which have high cerebellar d-serine levels (Figure 4). Therefore, the low accumulation of [^11^C]L-703,717 in forebrain regions may reflect the strong inhibition caused by the high level of endogenous d-serine. Similarly, the higher uptake of [^11^C]L-703,717 in the cerebellum might be due to reduced binding inhibition by d-serine [51].

### 2.3. Imaging Probes for the NR2B Negative Modulator Binding Site

**Figure 5:**
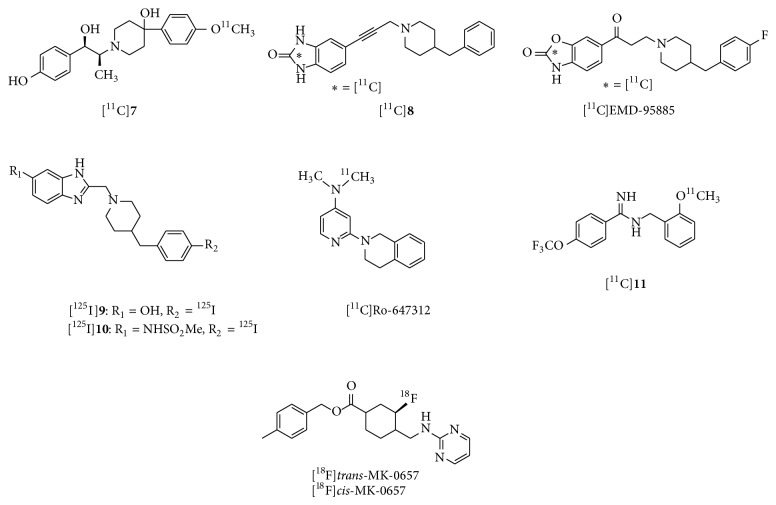
Chemical structure of imaging probes for NR2B negative modulator binding site of NMDARs.

**Figure 6:**
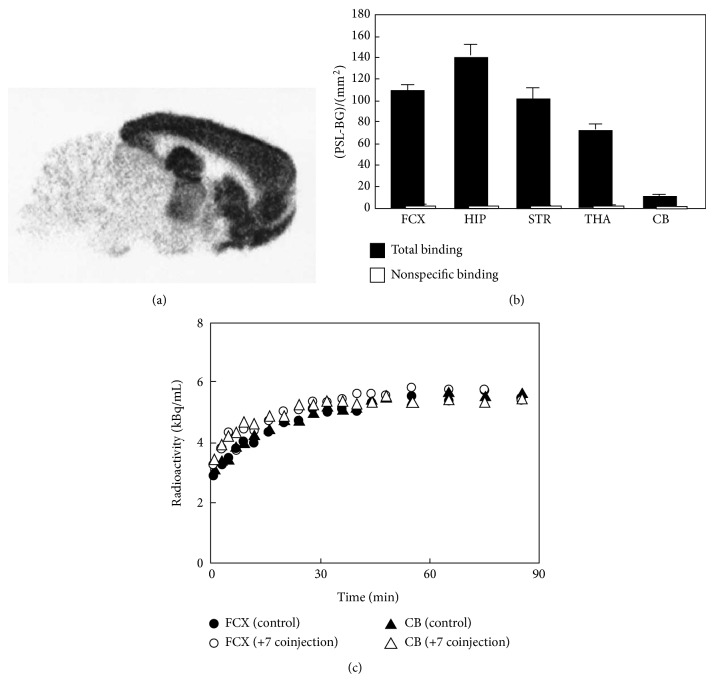
*In vitro *autoradiogram of [^11^C]**7 **(a) and quantified values of the autoradiogram in frontal cortex (*FTX*), hippocampus (*HIP*), striatum (*STR*), thalamus (*THA*), and cerebellum (*CB*) (b). Nonspecific binding was determined in the presence of (+) CP-101,606 (10 *μ*M). (c) Time radioactivity curves in the monkey brain after administration of [^11^C]**7**. Nonradioactive** 7** (2 mg/kg) was coinjected with [^11^C]**7 **into the same monkey [55].

NMDARs containing the NR2B subunit play a key role in various diseases, such as Parkinson's disease, Alzheimer's disease, and neuropathic pain. NR2B negative modulators have been developed for the treatment of these conditions [17, 52]. Ligands targeting NR2B, including ifenprodil, are thought to bind at the interlobe cleft of the NR2B subunit [53]. CP-101,606 is a potent NMDAR antagonist that is highly selective for NR2B subunit-containing receptors, with *K*_*d*_ values of 10 nM [54]. Because CP-101,606 was identified as a potent NR2B negative modulator (*K*_*i*_ = 10 nM), a ^11^C-labeled CP-101,606 derivative ([^11^C]**7**, Figure 5) was developed as a PET ligand for the NR2B subunit [55].* In vitro* binding of [^11^C]**7 **in rat brain slices was extremely high in the forebrain regions and very low in the cerebellum, with excellent specific binding (Figures 6(a) and 6(b)). This distribution pattern matched the NR2B subunit expression pattern [56]. However,* in vivo* studies in mice and monkeys demonstrated that this tracer showed homogeneous brain distribution and no specific binding of [^11^C]**7 **was observed (Figure 6(c)) [55]. A benzylpiperidine derivative, [^11^C]**8 **(Figure 5), has been developed as a selective high-affinity PET ligand for NR2B-containing NMDARs (IC_50_ = 5.3 nM). An* in vivo* study in rats showed poor brain uptake of [^11^C]**8** and a localization that was inconsistent with the NR2B expression pattern [57]. [^11^C]EMD-95885 (Figure 5), a benzylpiperidine derivative with a high affinity for NR2B (IC_50_ = 3.9 nM), has been synthesized and evaluated.* In vivo* experiments in rats showed 59-fold higher brain uptake of [^11^C]EMD-95885 than of [^11^C]**8**. Although [^11^C]EMD-95885 displayed homogeneous binding in brain tissues, a substantial reduction in brain uptake of [^11^C]EMD-95885 was observed in the presence of nonradioactive** 8** or ifenprodil, suggesting that some specific binding may occur in the brain. However, these blocking effects were observed in both NR2B-rich and NR2B-poor regions [58]. The benzimidazole derivatives,** 9** and** 10** (Figure 5), have been identified as having high affinity for the NR2B subunit, with *K*_*i*_ values of 7.3 nM and 5.8 nM, respectively. Both [^125^I]**9** and [^125^I]**10** showed localizations consistent with NR2B subunit expression in rat brain slices.* In vivo* studies in mice found moderate brain uptake of [^125^I]**9** and [^125^I]**10** and distribution that was inconsistent with known NR2B expression patterns. However, treatment with nonradioactive** 9 **or the NR2B ligand, [(±)-(*R* ^*^,  *S* ^*^)]-a-(4-hydroxyphenyl)-*β*-methyl-4-(phenylmethyl)-1-piperidine propanol (Ro 25–6981), caused 34% and 59% reduction in the brain/blood ratio of [^125^I]**9**, respectively. This tracer may therefore show partially specific binding to the NR2B subunit* in vivo *[59]. Further structural modification of** 9** may contribute to the development of more promising imaging probes for the NR2B subunit. [2-(3,4-Dihydro-1H-isoquinolin-2-yl)-pyridin-4-yl]–[^11^C]dimethylamine ([^11^C]Ro-647312, Figure 5) has been evaluated as a member of a different class of PET ligands with high affinity for the NR2B subunit (*K*_*i*_ = 8.0 nM). However,* in vivo *biodistribution of [^11^C]Ro-647312 was almost homogeneous in the brain [60]. Several benzylamidines, such as [^11^C]**11** (Figure 5), have been developed as high-affinity PET ligands for NR2B (*K*_*i*_ = 5.7 nM for** 11**).* In vitro*, [^11^C]**11** showed excellent specific binding and a similar localization to that of NR2B. However, [^11^C]**11** is an unsuitable imaging agent due to metabolic instability [61]. (*3S*,*4R*)-4-Methylbenzyl 3-fluoro-4-((pyrimidin-2-ylamino) methyl) piperidine-1-carboxylate (MK-0657) was developed as a highly potent NR2B antagonist (IC_50_ = 3.6 nM) for the treatment of neuropathic pain, Parkinson's disease, and major depression [17, 62]. Two radiofluorinated diastereoisomers of MK-0657 ([^18^F]*trans*-MK-0657 and [^18^F]*cis*-MK-0657, Figure 5) exhibited a localization pattern consistent with that of NR2B expression and very high specific binding for the NR2B modulator binding site. However, no further* in vivo* evaluations of these potential imaging agents have been reported [63].

## 3. mGluRs

mGluRs are widely expressed though the CNS and their activation leads to various effects on neuronal synaptic transmission via regulation of ion channels and signaling proteins. Dysregulation of mGluRs has been observed in various conditions affecting the CNS, such as anxiety [64], depression [65], Alzheimer's disease [66], schizophrenia [67], Parkinson's disease [68], and epilepsy [69]. Positive and negative modulators of mGluRs have therefore been developed for the treatment of these neurological diseases. Nuclear medicine imaging of mGluRs can be used for the investigation of a range of diseases, in addition to monitoring receptor occupancy by therapeutic agents. Thus, a considerable number of PET imaging probes for mGluRs have been reported. PET ligands for group I mGluRs (mGluR1 and 5) have been developed extensively and several of these have been confirmed as promising ligands in clinical studies. Recently, potential PET probes for mGluR2 (group II) have also been reported and have proceeded to phase I studies. No clinically useful PET ligands for group III mGluRs have been published, due to a lack of selectivity over other mGluRs.

### 3.1. Imaging Probes for Group I mGluRs

#### 3.1.1. Physiology of Group I mGluRs

Group I mGluRs (mGluR1 and mGluR5) are predominantly expressed in the postsynaptic neuron. Their activation leads to increased neuronal excitability and they are involved in modulation of synaptic plasticity at glutamatergic synapses. They are coupled to G_q_/G11 and upregulate inositol triphosphate and diacylglycerol levels via phospholipase C activation, triggering calcium mobilization, and activation of protein kinase C (PKC). In addition, group I mGluRs have been reported to be implicated in the mitogen-activated protein kinase (MAPK)/extracellular signal-regulated kinase (ERK) and mammalian target of rapamycin (mTOR)/p70 S6 kinase pathways, which can regulate synaptic plasticity [70]. Complementary expression of mGluR1 and mGluR5 has been observed in the rodent brain. mGluR1 are found extensively throughout the brain, but are highly expressed in the cerebellar cortex, hippocampus, and thalamus. mGluR5 expression has been observed in the cerebral cortex, hippocampus, accessory olfactory bulbs, and nucleus accumbens [71]. mGluR1 antagonists have shown promising anxiolytic and antidepressant effects, whereas positive modulators of mGluR1 have been reported to be useful for the treatment of schizophrenia. Negative modulators of mGluR5 can be effective in the treatment of anxiety, fragile X syndrome, chronic pain, and depression [72]. In contrast, positive modulators of this receptor have potential for the treatment of schizophrenia [73].

#### 3.1.2. Development of Imaging Probes for mGluR1

**Figure 7:**
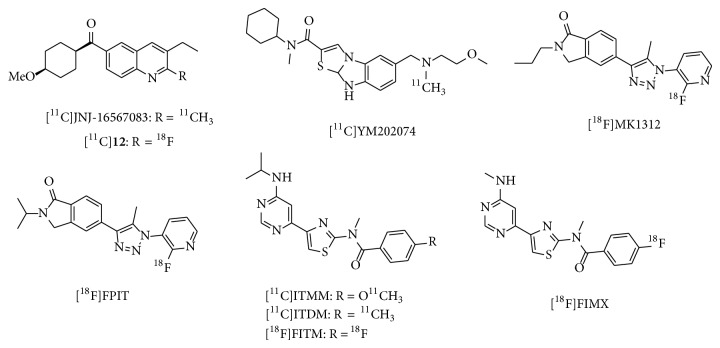
Chemical structure of imaging probes for mGluR1.

(3-Ethyl-2-[^11^C]methyl-6-quinolinyl)(*cis*-4-methoxycyclohexyl)methanone ([^11^C]JNJ-16567083, Figure 7) has been developed as a high-affinity, selective mGluR1 ligand (*K*_*i*_ = 4.41 nM for rat mGluR1, 13.3 nM for human mGluR1) [74].* Ex vivo* studies using this ligand showed good brain uptake and a localization pattern consistent with mGluR1 expression. In addition, over 80% of the accumulation of [^11^C]JNJ-16567083 in the cerebellum was blocked by selective mGluR1 antagonists, while treatment with a selective mGluR5 antagonist produced no marked inhibition of its binding, indicating selectivity for mGluR1. In PET studies, it showed high specific binding in regions expressing mGluR1, indicating that [^11^C]JNJ-16567083 is bound to mGluR1 in the living rat brain [74]. An ^18^F-labeled JNJ-16567083 derivative ([^18^F]**12**, Figure 7) was also reported to have a high affinity for mGluR1 (*K*_*i*_ = 1.77 nM for rat mGluR1 and 24.4 nM for human mGluR1). The* in vivo* localization of [^18^F]**12** in rats was similar to that of [^11^C]JNJ-16567083. The* in vivo* accumulation of [^18^F]**12** in the cerebellum was inhibited by pretreatment with a selective mGluR1 antagonist, but not by an mGluR2 antagonist (LY341495) or an mGluR5 antagonist (MPEP), indicating selectivity for mGluR1 in the living rat brain. However, PET studies in baboons found that [^11^C]JNJ-16567083 and [^18^F]**12** produced low brain signals, probably due to a lower receptor density in baboon brain than in rat brain [75]. These results suggested that less lipophilic and/or higher affinity mGluR1 ligands are necessary for successful imaging in the primate brain.

**Figure 8:**
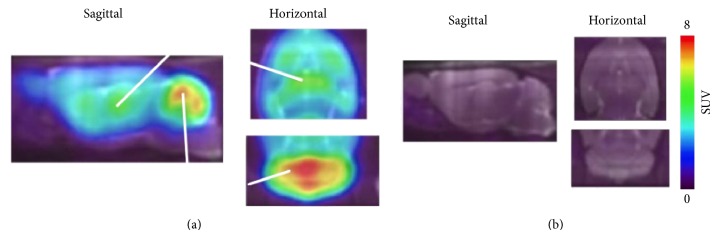
Sagittal PET images (0–60 min) of [^11^C]ITMM in wild-type (a) and mGluR1 knockout (b) mice brains [83].

**Figure 9:**
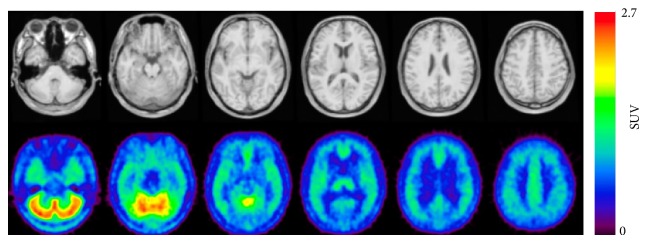
PET/MRI images of [^11^C]ITMM (40–60 min) obtained from 5 healthy human subjects. Upper: MR image and lower: averaged [^11^C]ITMM PET image [85].

*N*-Cyclohexyl-6-{[*N*-(2-methoxyethyl)-*N*-methylamino]methyl}-*N*-methylthiazolo [3,2-a]benzimidazole-2-carboxamide (YM-202074, Figure 7) has been reported as a high-affinity, selective mGluR1 ligand (*K*_*i*_ = 4.8 nM for rat mGluR1) with lower lipophilicity than JNJ-16567083 (log*D* = 2.7 versus 3.38). Although [^11^C]YM-202074 showed* in vitro* accumulation consistent with mGluR1 expression in the rat brain, PET studies in rats using this ligand demonstrated a low brain uptake and localization that was inconsistent with mGluR1-rich regions. These findings may be attributed to rapid ligand metabolism and the subsequent influx of radiometabolites into the brain [76]. 1-(2-Fluoro-3-pyridyl)-4-(2-propyl-1-oxoisoindoline-5-yl)-5-methyl-1H-1,2,3-triazole (MK-1312, Figure 7) has been developed as a potent mGluR1 ligand (IC_50_ = 4.3 nM for human mGluR1), with high selectivity and moderate lipophilicity (log*P* = 2.3). [^18^F]MK-1312 displayed similar* in vitro* localization to that of mGluR1 and highly selective binding. PET studies of this ligand in rhesus monkeys demonstrated rapid uptake kinetics, with no significant defluorination in brain. In addition, binding was inhibited by the mGluR1 antagonist, MK-5435, in a dose-dependent manner [77]. Although these results indicated that [^18^F]MK-1312 may be a promising PET ligand for mGluR1, no further clinical studies have been reported to date. 1-(2-Fluoro-3-pyridyl)-4-(2-isopropyl-1-oxoisoindoline-5-yl)-5-methyl-1H-1,2,3-triazole (FPIT, Figure 7), an MK-1312 derivative, has been reported as a selective mGluR1 ligand with an IC_50_ of 5.4 nM for human mGluR1 [77]. [^18^F]FPIT showed similar* in vitro* distribution to mGluR1 in both rat and monkey brains. In addition, its accumulation was selectively blocked by an mGluR1 ligand, indicating excellent specific binding (95%). PET/magnetic resonance imaging (MRI) studies of this ligand in rats and monkeys demonstrated a distribution that was consistent with that observed* in vitro*. Brain accumulation of [^18^F]FPIT was significantly inhibited by nonradioactive FPIT and by the mGluR1-selective ligand JNJ-16259865 [78]. Although [^18^F]FPIT has been demonstrated to be a prospective PET probe for mGluR1, further clinical studies have not yet been performed, probably due to the slow pharmacokinetics, and the influx of small levels of radiometabolites into the brain. 4-Fluoro-*N*-[4-[6-(Isopropylamino)pyrimidin-4-yl]-1,3-thiazol-2-yl]-*N*-methylbenzamide (FITM, Figure 7) was developed as a potent mGluR1 antagonist (IC_50_ = 5.1 nM) with high selectivity [79] and low lipophilicity (log*D* = 1.46) [80].* In vitro* and* ex vivo* binding of [^18^F]FITM matched the distribution of mGluR1, with high specific binding in mGluR1-rich regions [80]. [^18^F]FITM showed high brain uptake (>7% ID/g in mice) and metabolic stability, with 95% intact [^18^F]FITM detected in the rat brain 120 min after injection. Rat and monkey PET studies demonstrated a radioactive signal distribution consistent with that of the mGluR1, with very high specific binding. Kinetic analysis showed that the calculated *V*_*T*_ values of [^18^F]FITM were also consistent with the localization of mGluR1 [81]. PET studies of [^18^F]FITM in rat brain that included blocking experiments determined *B*_max⁡_ and *K*_*d*_ values in several brain regions with moderate mGluR1 expression, such as the thalamus, hippocampus, striatum, and cingulate cortex, consistent with the density of mGluR1 in these regions. However, because of its relatively slow kinetics, *B*_max⁡_ and *K*_*d*_ values of [^18^F]FITM could not be measured in the mGluR1-rich cerebellum [82]. Although these findings have shown that [^18^F]FITM is a prospective PET radiotracer for mGluR1, no further clinical PET studies have been reported in the literature. It is reported that [^18^F]FITM could be prepared in poor radiochemical yields (14 ± 3%), probably due to using the 4-nitrobenzamide precursor [80]. It is suggested that optimized methods for radiosynthesis of [^18^F]FITM should be developed.* N*-[4-[6-(Isopropylamino)pyrimidin-4-yl]-1,3-thiazol-2-yl]-4-methoxy*-N*-methylbenzamide (ITMM, Figure 7) has been reported to be a highly potent and selective PET probe for mGluR1, with a *K*_*i*_ value of 12.6 nM (rat brain homogenate) and a log*D* value of 2.57.* In vitro* binding of [^11^C]ITMM was consistent with mGluR1 distribution, with high specific binding in mGluR1-rich regions. A PET study in rats found that [^11^C]ITMM displayed high brain uptake, with the highest uptake (in the cerebellum) being over 3.0 of the SUV (standardized uptake value). The* in vivo *distribution of [^11^C]ITMM was consistent with the* in vitro* data. The heterogeneous localization of [^11^C]ITMM was abolished by treatment with nonradioactive ITMM and an mGluR1-selective ligand. In addition, a PET study of [^11^C]ITMM in the mGluR1 knockout mouse demonstrated quite low uptake and homogeneous distribution of radioactivity in the brain (Figure 8) [83]. Furthermore, [^11^C]ITMM showed reduced accumulation in the ischemic brain and treatment with the neuroprotective agent, minocycline, which may inhibit mGluR1 activation, abolished this decrease of mGluR1 in the brain [84]. Because [^11^C]ITMM has been demonstrated to be a promising PET tracer for mGluR1, the first human PET studies have been performed. [^11^C]ITMM uptake increased gradually in the cerebellar cortex and *V*_*T*_ in this brain region was 2.61, while *V*_*T*_ was 0.53 in the mGluR1-poor pons. The rank order of [^11^C]ITMM uptake was consistent with mGluR1 expression levels in the human brain (Figure 9). Because [^11^C]ITMM showed relatively low uptake in the brain regions with a modest expression of mGluR1, such as the thalamus, hippocampus, and cerebral cortex, the [^11^C]ITMM signal in regions outside the cerebellum could be difficult to assess and this would make it hard to examine localization changes in these regions [85]. Nevertheless, [^11^C]ITMM could be used to evaluate alterations in cerebellar mGluR1 under pathological conditions. Further clinical studies may be needed to assess the usefulness of [^11^C]ITMM as a PET ligand for quantification of mGluR1.

*N*-[4-[6-(isopropylamino)pyrimidin-4-yl]-1,3-thiazol-2-yl]-*N*-methyl-4-^11^C-methylbenzamide ([^11^C]ITDM, Figure 7), an analog of ITMM, also has high affinity for mGluR1 (*K*_*i*_ = 13.6 nM) and moderate lipophilicity (log*D* = 1.74). PET studies of [^11^C]ITDM in the monkey brain showed localization consistent with known mGluR1 expression, fast kinetics, and a very low level of binding in the mGluR1-poor pons [86]. After detailed kinetic studies, [^11^C]ITDM was considered superior to [^11^C]ITMM because of its higher regional *V*_*T*_ values in the monkey brain [87]. Global reduction of [^11^C]ITDM binding was observed in the mGluR1-expressing brain regions of the R6/2 mouse model of Huntington's disease. The change in the radioactive signal correlated with the expression of mGluR1 in the brains of these R6/2 mice [88]. Thus, [^11^C]ITDM has been confirmed as a promising PET ligand for monitoring changes in mGluR1 availability in the brain. To our knowledge, clinical PET studies with [^11^C]ITDM have not yet been published. [^18^F]4-Fluoro-*N*-methyl-*N*-(4-(6-(methylamino)pyrimidin-4-yl)thiazol-2-yl)benzamide ([^18^F]FIMX, Figure 7) has been shown to have a high affinity for mGluR1 (IC_50_ = 1.8 nM) with high selectivity against a wide range of other human receptors [79, 89]. [^18^F]FIMX was successfully synthesized by the radiofluorination of the diaryliodonium salts precursor. FIMX has moderate lipophilicity, with a log*D* value of 2.25. PET studies of [^18^F]FIMX in rhesus monkey demonstrated high BBB permeability and maximal uptake in the mGluR1-rich cerebellum (SUV = 5.3 at 12 min). The distribution of radioactivity in the brain matched mGluR1 expression, and accumulation was abolished by treatment with nonradioactive FIMX or the mGluR1-selective ligand JNJ-1625968553 (3 mg/kg, resp.). In contrast, an mGluR5-selective ligand (MTEP) did not affect [^18^F]FIMX binding in the monkey brain [89]. Apparently, [^18^F]FIMX is a prospective PET radioligand for imaging of mGluR1. Recently, an initial clinical PET imaging study using ^18^F-FIMX was performed. The mGluR1-rich cerebellum had the highest uptake (SUV; 1.8 at 120 min), whereas mGluR1-poor regions ranged from SUV values of 0.3–1 at 120 min [90]. PET imaging of patients with a range of CNS disorders using [^18^F]FITM, [^11^C]ITMM, [^11^C]ITDM, and [^18^F]FIMX could help to elucidate the relationship between these disorders and mGluR1.

#### 3.1.3. Development of Imaging Probes for mGluR5

**Figure 10:**
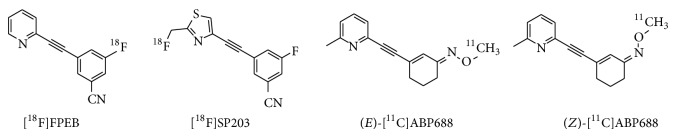
Chemical structure of imaging probes for mGluR5.

**Figure 11:**
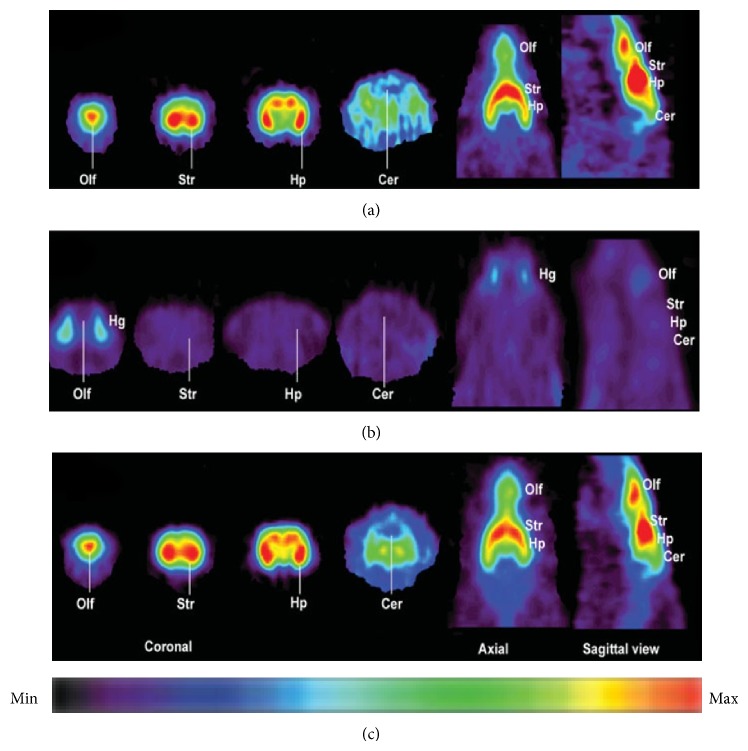
PET images of [^18^F]FPEB (20–25 min) in rat brain without drug treatment (a), treatment of mGluR5-selective ligand MTEP treatment (10 mg/kg) (b), and treatment of mGluR1-selective YM-298198 (10 mg/kg) [92].

Several diaryl alkyne derivatives have been reported as high-affinity, selective mGluR5 antagonists that could be used as* in vivo* imaging probes. 3-Fluoro-5-(2-pyridinylethynyl) benzonitrile (FPEB, Figure 10) has been reported to have an excellent affinity for mGluR5, with a *K*_*i*_ value of 0.2 nM (rat brain cortex) and a moderate log*P* value of 2.8. Autoradiography of rhesus monkey brain demonstrated high accumulation in mGluR5-rich regions, such as the cortex, caudate, putamen, amygdala, hippocampus, and most thalamic nuclei, and a low signal in the mGluR5-poor cerebellar layers. In addition, the heterogeneous binding of [^18^F]FPEB was abolished in the presence of the mGluR5-selective ligand, MPEP, indicating that the radiotracer bound specifically to mGluR5* in vitro *[91]. PET studies of [^18^F]FPEB in rhesus monkey and rat showed similar localization to mGluR5 and displacement by mGluR5 ligands (Figure 11) [91, 92]. Building on these promising preclinical studies of [^18^F]FPEB, an initial clinical PET study was performed to assess the usefulness of this radiotracer for PET imaging of mGluR5. The regional brain distribution of [^18^F]FPEB in the healthy adult brain matched the known expression of mGluR5 and showed high reproducibility, suggesting that [^18^F]FPEB is a suitable PET radioligand for mGluR5 in the human brain [93].

**Figure 12:**
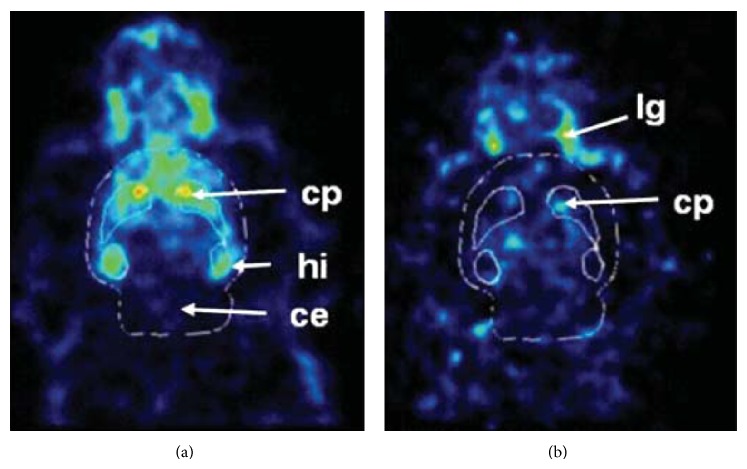
PET images of [^11^C]ABP688 (0–30 min) in control rats (a) and an mGluR5-selective ligand M-MPEP (1.0 mg/kg) treated rat [94].

**Figure 13:**
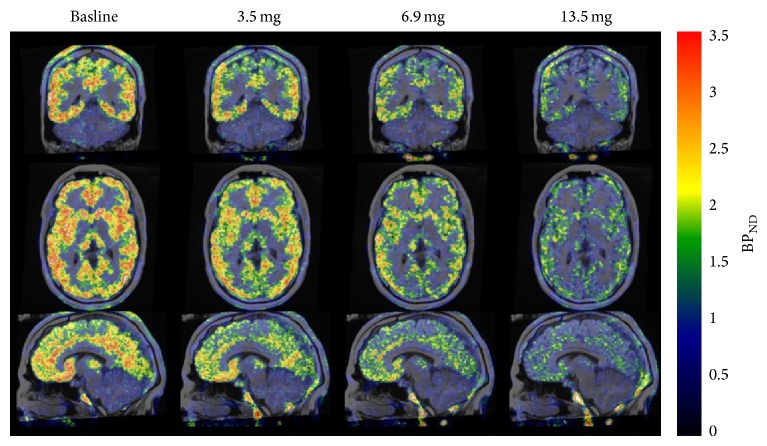
PET/MR images (0–63 min) showing the effect of AZD2066 on binding of [^11^C]ABP688 [96].

**Figure 14:**
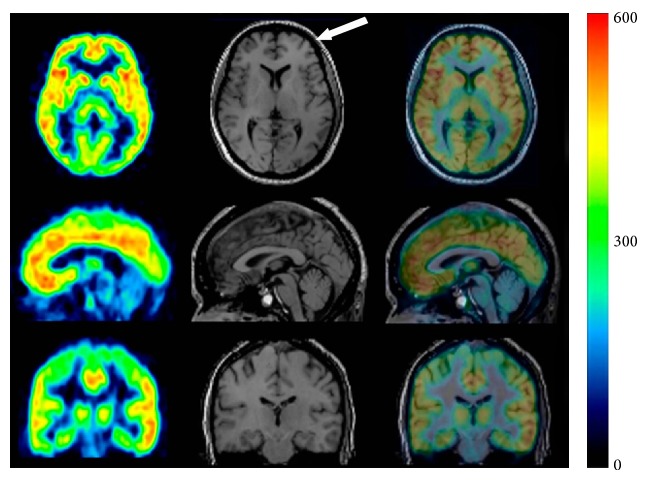
PET/MR images of [^18^F]SP203 (60–180 min) in healthy subject. Arrow points to subcutaneous fat [101].

3-(6-Methyl-pyridin-2-ylethynyl)-cyclohex-2-enone-O-^11^C-methyl-oxime ([^11^C]-ABP688, Figure 10) has been reported as a highly selective and potent radiotracer for the mGluR5 allosteric site, with a *K*_*d*_ value of 1.7 nM in rat brain homogenates.* Ex vivo *autoradiography of [^11^C]ABP688 demonstrated high accumulation of this radiotracer in mGluR5-rich brain regions, such as the cingulate cortex, striatum, and hippocampus. Treatment with M-MPEP (1 mg/kg), an mGluR5-selective antagonist, abolished the heterogeneous localization of [^11^C]ABP688. PET studies using [^11^C]ABP688 in normal rats demonstrated a similar distribution to that observed in* ex vivo* experiments (Figure 12). In addition, accumulation of this radiotracer was almost homogeneous in mGluR5-knockout mice, indicating that [^11^C]ABP688 was highly specific for mGluR5 in the living brain [94]. A clinical PET study of [^11^C]ABP688 in human brain demonstrated high accumulation in mGluR5-rich brain regions but low levels in the mGluR5-poor regions, consistent with the results of rat PET studies. Its specific distribution volume in the brain regions ranged from 5.45 (anterior cingulate) to 1.91 (cerebellum), and the rank order of these values was consistent with the known mGluR5 density. These results suggested that [^11^C]ABP688 could be used for PET quantification of mGluR5 in the human brain [95]. It has also been reported that [^11^C]ABP688 can be used for monitoring mGluR5 drug occupancy. AZD2066, a highly potent mGluR5-selective ligand, dose-dependently displaced [^11^C]ABP688 binding in the human brain (Figure 13). In this study, the dose of AZD2066 required to produce 50% mGluR5 occupancy was estimated to be 13.5 mg [96]. On the other hand, there are two geometrical isomers of [^11^C]ABP688 and the* E*-isomer has a much higher binding affinity for mGluR5 than the* Z*-isomer (*K*_*d*_ = 5.7 nM versus 140 nM). In a rat PET study of [^11^C]ABP688, the* E*-isomer showed high brain uptake and distribution that was consistent with mGluR5 expression. In contrast, the* Z*-isomer showed low brain retention and homogeneous binding throughout the brain [97]. Although [^11^C]ABP688 could be synthesized with an* E*-isomer to* Z*-isomer ratio of > 10 : 1 [94], it has been suggested that* E*-[^11^C]ABP688 should be used for reproducible imaging of mGlu5 receptors in clinical studies. 3-Fluoro-5-[2-[2-([^18^F]fluoromethyl)thiazol-4-yl]ethynyl]benzonitrile ([^18^F]SP203, Figure 10) has been developed as a negative allosteric modulator with excellent affinity (40 pM) for mGluR5. PET studies using [^18^F]SP203 demonstrated high uptake in mGluR5-rich regions and the signal was greatly diminished by treatment with the mGluR5-selective ligand MPEP (5 mg/kg) [98]. One disadvantage of [^18^F]SP203 is the ease of its defluorination by glutathione* S*-transferase in the rat brain [99]. However, because primates have much lower levels of glutathione* S*-transferase [100], initial PET studies were conducted with [^18^F]SP203 in healthy human subjects. PET images showed high brain uptake of [^18^F]SP203 (Figure 14) with little defluorination and brain uptake could be calculated as *V*_*T*_ in humans. [^18^F]SP203 had high BBB permeability (% SUV; ~580) and the rank order of *V*_*T*_ values in the brain regions was as follows: neocortex (20–26) > thalamus (15) ≒ cerebellum (14), consistent with the levels of mGluR5 in the human brain [101]. Therefore, despite the issue of defluorination, ^18^F-SP203 can be used for quantification of mGluR5 by PET.

#### 3.1.4. mGluR5-Selective PET Imaging in Diseases

**Figure 15:**
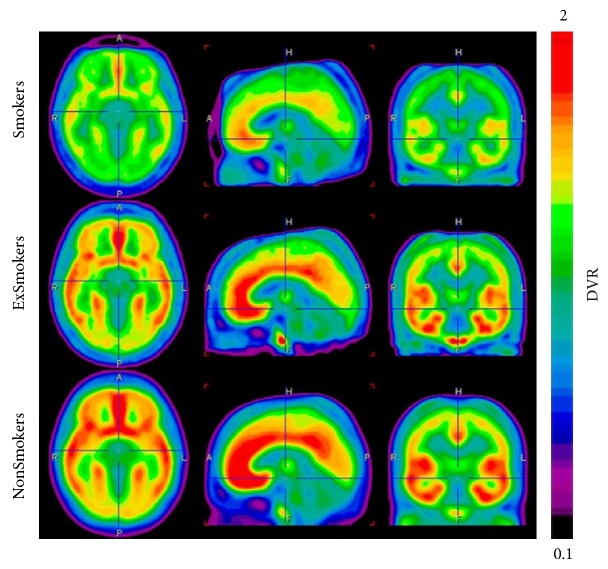
PET Images of the average mGluR5 DVR (0–40 min) using [^11^C]ABP688 in the three diagnostic groups (*n* = 14) [107].

Cocaine exposure has been reported to reduce mGluR5 expression in the rodent brain [102]. Clinical PET studies were conducted with [^11^C]ABP688 in cocaine-addicted participants, in comparison with healthy control subjects. [^11^C]ABP688 binding in the striatum was reduced by approximately 20% in cocaine-addicted participants [103]. Another clinical PET study with [^11^C]ABP688 demonstrated that mGluR5 availability in cocaine-dependent subjects was inversely proportional to the duration of cocaine abstinence [104]. These studies indicated that distribution of mGluR5 was decreased in the striatum of the living brain in cocaine-abstinent individuals, information that could inform novel strategies for the control of cocaine addiction* via *mGluR5. Preclinical studies have demonstrated that mGluR5 antagonists potently reduced self-administration of nicotine [105, 106]. In a PET study, a significant reduction in [^11^C]ABP688 binding was observed in the gray matter of smokers (Figure 15), indicating that mGluR5 could be a potential target for the treatment of nicotine dependence [107]. mGluR5 have been suggested to be involved in the pathophysiology of epilepsy [108, 109]. PET studies using [^11^C]ABP688 revealed lower levels of mGluR5 binding in the hippocampus and amygdala in rat models of chronic epilepsy, as compared to a control group. Therefore, PET imaging of mGluR5 in the temporal and spatial regions could detect dysregulated glutamatergic networks during epileptogenesis [110]. mGluR5 may also be linked to the pathophysiology of major depression [65]. Clinical PET studies using [^11^C]ABP688 showed reduced mGluR5 binding in the cortical regions, thalamus, and hippocampus of patients with depression. Furthermore, mGluR5 availability in the hippocampus decreased with the increased severity of this disease, suggesting that changes in the availability of mGluR5 may provide a potential biomarker for the diagnosis of depression [111].

### 3.2. Imaging Probes for Groups II and III mGluRs

#### 3.2.1. Physiology of Groups II and III mGluRs

Groups II (mGluR2 and mGluR3) and III (mGluR4, mGluR6, mGluR7, and mGluR8) mGluRs are primarily localized within presynaptic regions and involved in the inhibition of neurotransmitter release [70]. Groups II and III mGluRs are coupled to G_i_/G_o_ and downregulate cAMP levels via inhibition of adenylate cyclase. These events activate the MAPK and phosphatidylinositol 3-kinase (PI3) pathways, which regulate synaptic transmission. mGluR2 has been predominantly observed in the cerebellar cortex and olfactory bulb, while mGluR3 has been found widely throughout the brain. mGluR4 is predominantly expressed in the cerebellum. The expression of mGluR6 is restricted to the retina, whereas mGluR7 has a widespread distribution in the brain. mGluR8 is mainly expressed in the olfactory bulbs, olfactory nucleus, piriform cortex, entorhinal cortex, and medulla oblongata [71]. Agonists of group II mGluRs may be useful in the treatment of anxiety disorders and schizophrenia [73]. mGluR2/3 are involved in the pathophysiology of schizophrenia [112]. Group III mGluRs are attractive targets for the treatment of Parkinson's disease [113]. mGluR8 is implicated in drug addiction mechanisms [114].

#### 3.2.2. Development of Imaging Probes for Group II mGluRs

**Figure 16:**
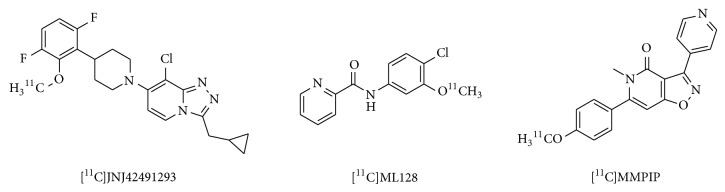
Chemical structure of imaging probes for groups II and III mGluRs.

8-Chloro-3-(cyclopropylmethyl)-7-[4-(3,6-difluoro-2-methoxyphenyl)-1-piperidinyl]-1,2,4-triazolo[4,3-a]pyridine ([^11^C]JNJ42491293, Figure 16) has been reported to act as a potent positive allosteric modulator for mGluR2, with an IC_50_ of 9.2 nM for the human mGluR2 and a high selectivity against other mGluRs.* In vivo* biodistribution and PET studies in rats demonstrated a high brain uptake of [^11^C]JNJ42491293, with a signal matching the known distribution of mGluR2 that was displaced by JNJ42153605, a selective mGluR2 agonist, in all brain regions. [^11^C]JNJ42491293 may be a promising PET radiotracer for the mGluR2 allosteric binding site [115]. PET studies using [^11^C]JNJ42491293 in healthy male subjects demonstrated high brain uptake, which reached a maximum 30 min after treatment. Considerable [^11^C]JNJ42491293 distribution was observed in the striatum and cerebellum, consistent with known mGluR2 expression patterns. [^11^C]JNJ42491293 has been demonstrated to be a prospective PET radioligand for monitoring mGluR2 availability in the human brain [116].

#### 3.2.3. Development of Imaging Probes for Group III mGluRs

*N*-(Chloro-3-methoxyphenyl)-2-picolinamide (ML128, Figure 16) has been developed as a positive allosteric modulator of mGluR4 with an EC_50_ of 110 nM for rat mGluR4 [117]. [^11^C]ML128 has been synthesized and evaluated as a PET imaging probe for mGluR4. In rat PET studies, [^11^C]ML128 showed high BBB penetration and localization to the hippocampus, striatum, thalamus, olfactory bulb, and cerebellum, consistent with the distribution of mGluR4. Treatment with an mGluR4 modulator led to some reduction (22–28%) in [^11^C]ML128 binding in the brain. Because this radiotracer exhibited fast washout within 20 min throughout the brain, structural modification will be necessary to provide useful* in vivo* imaging probes for mGluR4 [118]. MMPIP (6-(4-methoxyphenyl-5-methyl-3-pyridin-4-ylisozazolo[4,5-c]pyridine-4(5H)-one) is an allosteric mGluR7 antagonist with a *K*_*b*_ of 30 nM [119]. [^11^C]MMPIP (Figure 16) has been synthesized and evaluated as a PET radioligand for mGluR7. Although [^11^C]MMPIP showed specific* in vitro *binding in rat brain, PET studies of this radioligand in rats demonstrated low brain uptake and no specific localization to sites of mGluR7 expression.

## 4. Conclusion and Perspectives

Despite considerable efforts, no radioligands are currently available for human* in vivo* PET or SPECT imaging of NMDARs. There are three potential target sites for imaging agents; the channel blocker site, the glycine site, and the NR2B negative modulator site. [^123/125^I]CNS 1261 is a potential imaging agent acting at the channel blocker site. However, this ligand requires further research to address its input function alteration in pathological state metabolic instability and potentially nonspecific binding. There are no radioligands for glycine site or the NR2B negative modulator site that have shown enough promise to warrant clinical studies. Although several ligands showed localization patterns consistent with NMDAR expression and high specific binding* in vitro*, they did not show these characteristics* in vivo*. This discrepancy might be due to the dynamic properties of NMDARs. It is known that NMDAR structure is altered by binding of endogenous or exogenous ligands [1]. Importantly, the number of NMDARs on the cell surface is changed by tyrosine phosphorylation, cysteine palmitoylation of the NMDAR C-terminus, and by an alteration in subunit composition [120–122]. In addition to developing novel classes of imaging probes for NMDARs, further* in vivo* imaging studies of NMDAR models should be performed to clarify the discrepancies between the findings of* in vitro* and* in vivo* studies.

In contrast to the situation with NMDARs, several promising PET probes for mGluRs have been developed successfully. PET radioligands for mGluR1, such as [^18^F]FITM, [^11^C]ITMM, [^11^C]ITDM, and [^18^F]FIMX, have proved useful in clinical PET studies. PET imaging studies of mGluR5 with selective radiotracers ([^18^F]FPEB, [^18^F]SP203, and [^11^C]ABP688) have enabled evaluation of mGluR5 availability in relevant disease states. [^11^C]JNJ42491293 has been developed as a clinically useful prospective PET probe for mGluR2. Further PET investigations of patients with various mGluR-related diseases could make it possible to resolve the roles of mGluRs in these disorders, monitor mGluR activity, and quantify mGluR occupancy by therapeutic agents. As no clinically useful PET ligands for group III mGluRs have yet been identified, continued efforts should be made to develop high-affinity and selective ligands for these receptors.
